# Circulating miRNAs as Epigenetic Mediators of Periodontitis and Preeclampsia Association

**DOI:** 10.1155/2022/2771492

**Published:** 2022-07-11

**Authors:** Wangmeng Zhang, Qishan Wu, Juan Su, Wenwen Wang, Xudong Zhao, Ping Cao

**Affiliations:** ^1^Department of Obstetrics, The Affiliated Tai'an City Central Hospital of Qingdao University, Tai'an, 271000 Shandong Province, China; ^2^Department of Stomatology, The Affiliated Tai'an City Central Hospital of Qingdao University, Tai'an, 271000 Shandong Province, China; ^3^Department of Obstetric and Gynecologic Ultrasound, The Affiliated Tai'an City Central Hospital of Qingdao University, Tai'an, 271000 Shandong Province, China; ^4^Department of Geriatrics, The Affiliated Tai'an City Central Hospital of Qingdao University, Tai'an, 271000 Shandong Province, China

## Abstract

**Objective:**

Periodontal disease has been associated with pregnancy complications including preeclampsia. This bioinformatic study is aimed at investigating the possible role of circulating microRNAs (miRNAs) as mediators of the association between maternal periodontal disease and preeclampsia.

**Methods:**

Peripheral blood miRNA profiles of periodontitis and controls were sought from Gene Expression Omnibus (GEO), and differential expression analysis was performed. Experimentally validated circulating miRNAs associated with preeclampsia were determined from the Human MicroRNA Disease Database (HMDD v3.0). Venn diagrams were drawn to identify shared circulating differential miRNAs (DEmiRNAs). Significantly enriched target genes, KEGG pathways, and Gene Ontology (GO) terms for the set of shared DEmiRNA were predicted using miRNA enrichment analysis and annotation tool (miEAA v 2.0). Additionally, the shared DEmiRNA-enriched target genes were analyzed for enriched WikiPathways, BioCarta metabolic pathways, and tissue proteins in the human proteome map.

**Results:**

Among 183 circulating DEmiRNA in periodontitis and 60 experimentally validated miRNA in preeclampsia, 9 shared DEmiRNA were identified. The top among 32 overrepresented target genes included MAFB, PSAP, and CDK5RAP2, top among 14 enriched KEGG pathways were renin-angiotensin system and graft-versus-host disease, and that among enriched 44 GO profiles included “positive regulation of epidermal growth factor-activated receptor activity” and “sequestering of calcium ion.” In the overrepresented target gene set, among 10 enriched WikiPathways, the top included “NAD metabolism, sirtuins, and aging” and “regulation of Wnt/B-catenin signaling by small molecule compounds” and PPAR-related mechanisms was top among 13 enriched BioCarta metabolic pathways.

**Conclusion:**

A circulating 9-DEmiRNA set was significantly linked to both periodontitis and preeclampsia. Enrichment analysis identified specific genes, pathways, and functional mechanisms, which may be epigenetically altered and thereby mediate the biological association of periodontitis and preeclampsia.

## 1. Introduction

Periodontitis is a chronic, multifactorial inflammatory disease affecting the tooth-supporting structures and a primary cause of tooth loss [1]. Globally, periodontitis prevalence is estimated to affect 20 to 50 percent adults [2]. Periodontitis is associated with multiple systemic diseases and metabolic disorders including obesity, cardiovascular disease, and diabetes, among others [3]. Apart from shared risk factors, systemic inflammatory burden arising from chronic periodontal infection is considered central to the risk increment for systemic diseases attributed to periodontal disease [3–5]. Hypertensive disorders of pregnancy are highly prevalent and pose risk for maternal and neonatal mortality and morbidity. These include preeclampsia, defined as occurrence of gestational hypertension and proteinuria after 20 weeks, which is a major risk factor for preterm labour and low birth weight [6, 7]. A number of risk factors for preeclampsia have been established and include preexisting hypertension, diabetes mellitus, obesity, and multiparity among others [8, 9]. Systemic inflammation triggered by various infectious diseases can also contribute to the development of preeclampsia [10]. Pregnancy is associated with a physiological activation of the immune system [11]. Infectious diseases can perturb the delicate balance of innate and cellular immune responses by activation of pathogen-associated molecular patterns and downstream proinflammatory effects, ultimately contributing to deregulated inflammatory responses affecting placental tissue, endothelial function, and maternal circulation, leading to preeclampsia [12, 13].

Among infectious diseases, maternal periodontitis has been linked to pregnancy complications and adverse outcomes including gestational hypertension, preeclampsia, preterm delivery, and low birth weight [14–16]. Evidence also supports some clinical benefits of treating periodontal disease in pregnant women in terms of reduced incidence of pregnancy complications [17]. At the same time, the molecular mechanisms underlying the contribution of periodontitis to preeclampsia risk or pathology are not well understood. Emerging evidence has demonstrated the role of epigenetic deregulation via circulating miRNA activity in the pathogenesis of both periodontitis and preeclampsia [18–20]. MicroRNA (miRNA) are small noncoding RNA that typically serve as transcriptional repressors by attenuating mRNA transcription by mechanisms such as deadenylation and degradation of mRNA [21]. miRNA function is central to the maintenance of physiological functions, and altered miRNA profiles are associated with a wide variety of pathological states including cancer and inflammatory and metabolic disorders [22]. Circulating miRNAs may reflect both local aberrations in disease and also may have the potential to exert remote effects, thereby mediating systemic disorders. In the present bioinformatic study, we aimed to investigate the potential role of circulating miRNAs in mediating the association of periodontitis with preeclampsia.

## 2. Materials and Methods

### 2.1. Datasets for Circulating miRNAs in Periodontitis and Preeclampsia and Shared Differentially Expressed miRNA (DEmiRNA)

The Gene Expression Omnibus (GEO) dataset GSE31568 containing whole blood “miRNome” or miRNA expression profiles from multiple cancer and noncancer diseases was downloaded and filtered to retain 18 periodontitis [23] and 70 healthy control samples. Differential expression analysis was performed using the R package “limma” in GEO2R [24]. In brief, the expression data were log_2_ transformed, normalized, and assigned precision weights using “vooma” and miRNA with FDR-adjusted *p* value < 0.05 and logFC > 0 were considered as circulating DEmiRNA in periodontitis. The Human MicroRNA Disease Database (HMDD v3.0) [25] was searched for experimentally validated miRNA in preeclampsia and filtered to retain circulating miRNA among these. A Venn diagram was drawn using the two sets to identify circulating DEmiRNA common to both periodontitis and preeclampsia and utilized for subsequent analysis.

### 2.2. Overrepresentation Analysis of Target Genes, Pathways, and Gene Ontology (GO) Terms

Overrepresentation analysis of the shared circulating DEmiRNA set was performed using the “miRNA enrichment analysis and annotation tool” (miEAA 2.0) web interface (https://ccb-compute2.cs.uni-saarland.de/mieaa2) [26]. The species was selected as “Homo sapiens,” and overrepresented target genes utilizing the miRTarBase database, KEGG pathways, GO terms using the miRWalk database were determined with the selection criteria set as Benjamini-Hochberg FDR-adjusted *p* value < 0.5 and 2 minimum required hits per subcategory.

### 2.3. Functional Enrichment Analysis of the Overrepresented Target Genes

The set of significantly overrepresented genes by the shared DEmiRNA identified in the previous step was subjected to functional enrichment analysis using the webtool “Enrichr” (https://maayanlab.cloud/Enrichr/) [27]. The WikiPathways 2021 human database [28] was utilized for pathway enrichment analysis, and the BioCarta 2016 database [29] was utilized for enrichment analysis of metabolic and signaling pathways, with Benjamini-Hochberg FDR-adjusted *p* value < 0.05 considered as significant.

## 3. Results

### 3.1. Shared DEmiRNA between Periodontitis and Preeclampsia

**Figure 1:**
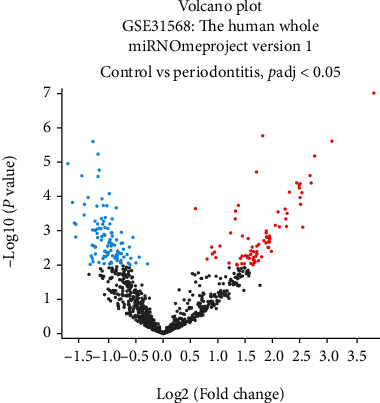
Volcano plot depicting circulatory DEmiRNA in periodontitis (GSE31568 human whole miRNOme dataset).

A total of 183 circulating DEmiRNA were determined in periodontitis with FDR-adjusted *p* value < 0.05 and logFC > 0 (Figure 1, Supplementary Table S1). 60 experimentally validated circulatory miRNA in preeclampsia were identified in the Human MicroRNA Disease Database (HMDD v3.0) (Supplementary Table S2).

**Figure 2:**
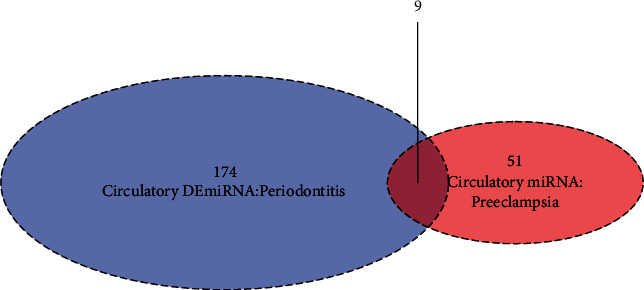
Venn diagram showing the overlap of circulatory DEmiRNA in periodontitis with experimentally validated circulating miRNA in preeclampsia.

Construction of Venn diagram to determine the overlap yielded 9 shared circulatory DEmiRNA (Table 1, Figure 2). Hsa-mir-122 and Hsa-mir-152 were downregulated significantly in periodontitis and in preeclampsia while Hsa-mir-130b displayed opposite patterns in both diseases.

### 3.2. Overrepresented Target Genes, Pathways, and Gene Ontology (GO) Terms by the Shared DEmiRNA

32 target genes overrepresented in the shared DEmiRNA are listed in Table 2.

**Figure 3:**
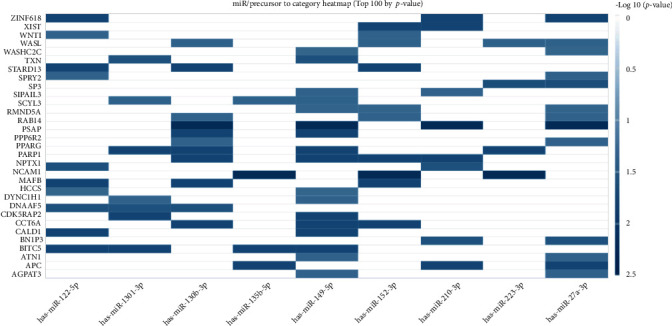
Target genes significantly overrepresented in the shared DEmiRNA associated with periodontitis and preeclampsia presented as a heat map. Columns represent the input shared DEmiRNA and rows represent the target genes. Color of the cells represents –log10 *p* values.

**Figure 4:**
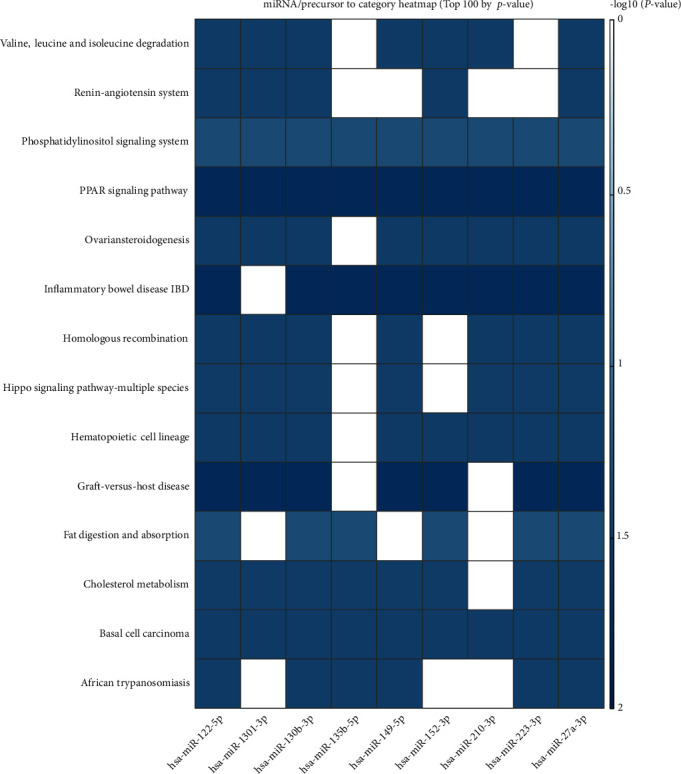
KEGG pathways significantly overrepresented in the shared DEmiRNA associated with periodontitis and preeclampsia presented as a heat map. Columns represent the input shared DEmiRNA and rows represent the target genes. Color of the cells represents –log_10_*p* values.

**Figure 5:**
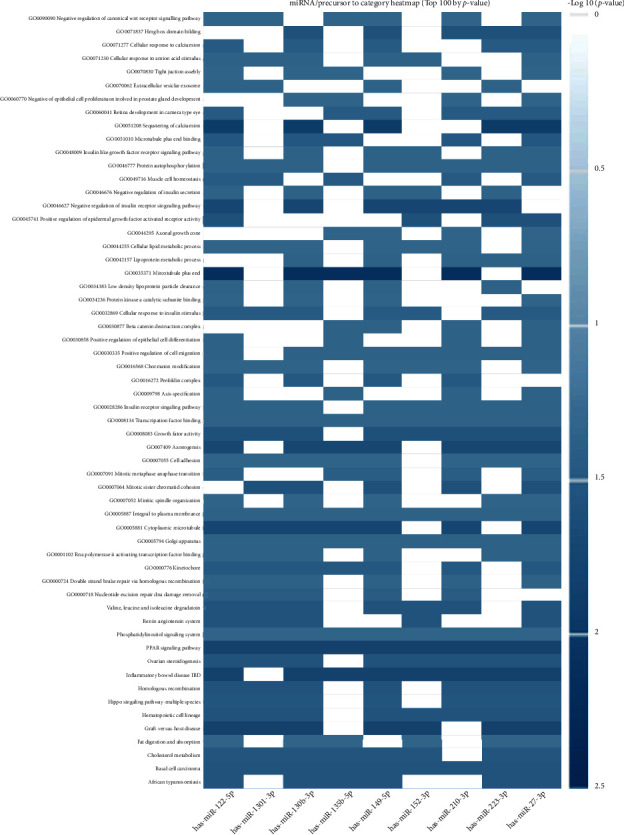
GO terms significantly overrepresented in the shared DEmiRNA associated with periodontitis and preeclampsia presented as a heat map. Columns represent the input shared DEmiRNA and rows represent the target genes. Color of the cells represents –log_10_*p* values.

The top 10 overrepresented target genes included MAFB, PSAP, CDK5RAP2, XIST, CALD1, PPP6R2, APC, ZNF618, STARD13, and HCCS (Table 2, Figure 3). The top 5 overrepresented KEGG pathways included renin-angiotensin system, graft-versus-host disease, African trypanosomiasis, fat digestion and absorption, and inflammatory bowel disease (IBD) (Table 3, Figure 4). The top 5 overrepresented GO terms included GO0045741 positive regulation of epidermal growth factor-activated receptor activity, GO0051208 sequestering of calcium ion, GO0016272 prefoldin complex, GO0035371 microtubule plus end, and GO0030877 beta catenin destruction complex (Table 4, Figure 5).

### 3.3. Functional Enrichment Analysis of the Overrepresented Target Genes

**Figure 6:**
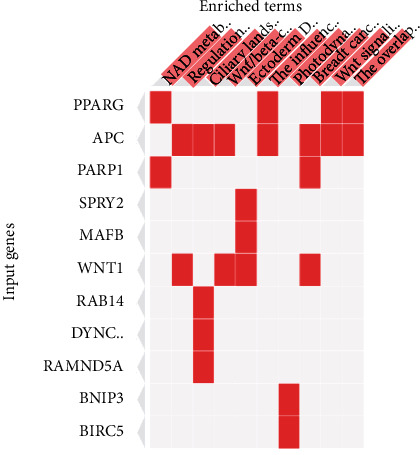
WikiPathways (2021 human) significantly enriched in the overrepresented target genes sorted by combined scores (*p* value and *z* score) as heat maps. Enriched terms presented in columns and input genes as rows. Colored cells indicate a gene is associated with the indicated pathway.

**Figure 7:**
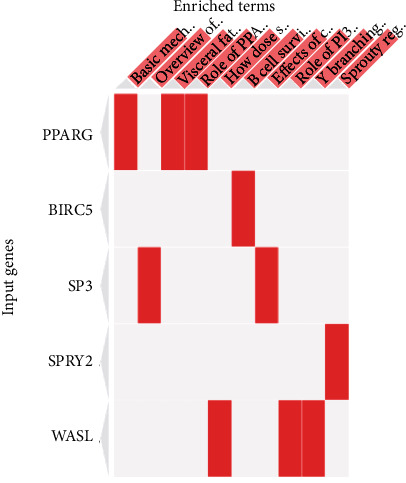
BioCarta (2016) pathways significantly enriched in the overrepresented target genes sorted by combined scores (*p* value and *z* score) as heat maps. Enriched terms presented in columns and input genes as rows. Colored cells indicate a gene is associated with the indicated pathway.

The significantly enriched WikiPathways are represented in Table 5 and Figure 6. The top 3 enriched pathways included NAD metabolism, sirtuins, and aging (WP3630), regulation of Wnt/B-catenin signaling by small molecule compounds (WP3664), and ciliary landscape (WP4352). The significantly enriched BioCarta metabolic and signaling pathways are represented in Table 6 and Figure 7, which included basic mechanism of action of PPARa, PPARb(d), and PPARg and effects on gene expression, overview of telomerase RNA component gene, and visceral fat deposits and the metabolic syndrome as the top 3 enriched metabolic pathways.

## 4. Discussion

The present study focused on circulating miRNAs as molecular mediators of the association between periodontitis and preeclampsia. miRNAs are considered important regulators of maternal-fetal cross-talk, circulating in the extracellular fluids [30] and packed within extracellular vesicles produced by the placenta [31]. miRNAs are critical modulators of endotoxin tolerance at this interface [32]. Placenta-derived exosomal miRNAs are considered to modulate the tolerance to LPS [33], which could be a mechanism linking vascular disorders in pregnancy with periodontitis. Here, we explored experimentally validated circulating miRNA associated with preeclampsia that also showed aberrant expression in periodontitis. Nine shared DEmiRNAs were determined. Among these, miRNA 122 and miRNA 152 showed downregulation in periodontitis and were also experimentally validated as downregulated in preeclampsia. miRNA 122 is the dominantly expressed miRNA in the liver and is found to be released in circulation during sepsis, leading to downstream complement activation and cytokine release [34, 35]. miRNA 122 is implicated in the epigenetic modulation of energy metabolism and angiogenesis [36]. A lower circulating miRNA 122 was shown to follow preeclampsia in the long term [37], while others have reported its elevation in preeclamptic placental tissues [38]. miRNA 122 is also found to be secreted by LPS-induced neutrophils [39]. Aberrant miRNA 152 has been reported in placental tissues in pregnancy complications [40]. miRNA 152 is also reported to regulate the innate immune response and antigen-presenting capacity of dendritic cells [41] and altered in circulating mononuclear cells stimulated by LPS [42]. It is plausible that the shared DEmiRNAs mediate deleterious periodontitis-associated endotoxin tolerance and inflammatory pathways active at the maternal-fetal interface, which is known to contribute to preeclampsia [33]. miRNA 130 was evident as highly significant and has been documented to promote vasoconstriction in pulmonary hypertension via deregulation of vasoactive mediators such as endothelin-1 [43]. miRNAs play important roles in mediating endotoxin tolerance at placental tissue via feedback loops at TLR receptors, and miRNAs packaged in extracellular vesicles translocate to remote sites including trophoblasts and maternal tissues effecting proinflammatory mechanisms [33]. Perturbations in circulatory miRNAs can thus deregulate the homeostatic mechanisms in these tissues.

To further explore potential molecular mechanisms in this context, we performed functional enrichment analysis of the shared miRNA set. MAFB and PSAP genes showed the highest overrepresentation. MAFB gene polymorphisms are associated with lipid levels, coronary disease, and atherosclerosis [44]. MAFB encodes for the lysosomal protein prosaposin, and its expression increases in response to stimuli promoting macrophage M2 state polarization and cholesterol efflux [45]. Periodontitis risk attributed to the immune receptor gene SIGLEC5 is shown to act via impaired MAFB binding [46]. PSAP encodes for prosaposin, which is linked to mTOR signaling and its macrophage expression is reported in atherosclerosis-associated inflammation [47]. Posttranscriptional regulation of placental PSAP has also been implicated in early-onset preeclampsia [48]. The highest observed to expected ratio value was seen for CDK5RAP2, a regulator of CDK5 (cyclin-dependent kinase 5) activity, which is implicated in preeclampsia via the regulation of Nestin, a cytoskeleton protein expressed in podocytes that serve as barriers to protein leakage [49]. Cyclin-dependent kinases are also considered as initiators of proinflammatory mediators via stimulating transcriptional activity including that of nuclear factor kappa B [50]. Among these, CDK9 has been found to mediate necroptosis in periodontitis [51].

The top two overrepresented KEGG pathways included renin-angiotensin system (RAS) and graft-versus-host disease. Gene polymorphisms in the RAS components modulate inflammation which have been associated with severe periodontitis [52] and a local tissue-RAS system has been shown to operate in periodontal tissue [53], but the effects on systemic RAS are unclear. In preeclampsia, a dysregulated RAS is considered to play a key role in pathogenesis by release of placental renin, other RAS proteins, and miRNA [54]. The perturbation of the graft-versus-host disease (GVHD) pathway in periodontitis is not well investigated but clinically periodontitis is shown to act as a risk factor for GVHD [55]. The pathophysiology of preeclampsia has been considered similar to that of autoimmune diseases including GVHD [56], and a number of investigations have shown GVHD-associated genetic and epigenetic features as significant markers in preeclampsia [57, 58]. Notably, integrated bioinformatics analysis has shown molecular subtypes of preeclampsia, whereby one subtype was marked by overrepresentation of autoimmune pathways including GVHD [59]. In addition, the inflammatory bowel disease (IBD) pathway was also evident as deregulated. Women with IBD have been reported to have higher risk of adverse pregnancy outcomes and severe preeclampsia [60]. These findings support a hypothesis that periodontitis-associated circulatory epigenetic alterations may contribute to an autoimmune component implicated in preeclampsia. The top overrepresented GO term was positive regulation of epidermal growth factor-activated receptor activity. Periodontitis-linked inflammation is shown to activate epidermal growth factor binding capacity [61]. Excessive epidermal growth factor in preeclamptic placental tissue has been identified as a mechanism responsible for deregulating the antiangiogenic factor, sFlt-1 [62]. Aberrant calcium transport by the placental syncytiotrophoblast has been implicated in the pathophysiology of preeclampsia [63]. However, the potential mechanisms of periodontitis induced alteration of placental calcium transport remain to be investigated.

In the following steps, enrichment analysis of the overrepresented target gene set was conducted. The NAD metabolism, sirtuin, and aging pathway was notably enriched. Lowering of multiple sirtuins has been demonstrated in preeclamptic placental syncytiotrophoblast and considered to represent a response to inflammatory and stress stimuli leading to lower energy reserve [64]. Periodontal therapy has been found to increase sirtuin-1 alongside lowering of systemic inflammatory markers [65]. These findings support the notion that miRNA-mediated deregulation of sirtuins and related metabolic signaling could comprise a key molecular pathway linking periodontal inflammation and preeclampsia. Several Wnt-related pathways were enriched among the target genes of the shared DEmiRNA. Wnt signaling is implicated as an important pathway in preeclampsia contributing to invasion of the trophoblast [66]. LPS from the periodontal pathogen P. gingivalis is shown to activate Wnt signaling pathways in the apical papilla [67] suggesting its role as a mechanistic mediator in the association of the two diseases. Significantly enriched BioCarta metabolic and signaling pathways included multiple PPAR-associated pathways. A critical role of PPAR signaling in preeclampsia has been demonstrated, and PPAR antagonist was found to induce a preeclamptic state [68]. Decreased PPAR-*γ* levels have also been demonstrated in periodontitis [69]. Therefore, epigenetic deregulation of PPAR signaling could be a linkage mechanism between periodontitis and preeclampsia.

These findings highlight several putative key mechanistic pathways that are epigenetically deregulated in a periodontitis state and can thereby contribute to preeclampsia development. However, the present study has limitations. A major limitation is the lack of validation experimental data from animal or cell models or clinical samples. The DEmiRNAs, target genes, and signaling pathways necessitate verification via targeted experimental approaches. Other limitations include the limited number of samples and absence of sampling population diversity, which may curtail the generalizability of these findings. Availability of large datasets with multiple metadata layers can permit more granular and confident understanding of the range of epigenetic mechanisms involved in this context. Furthermore, shared host genetic predisposition including plasminogen activator inhibitor-1 polymorphism [70, 71] and environmental risk factors like smoking [15] or obesity [72, 73] may underlie the association of preeclampsia and periodontitis, which were not accounted for in the current study design. In addition, in the present design, experimentally validated miRNA dataset was used only for preeclampsia and not for periodontitis, which may limit the validity of these findings. As circulating miRNAs are known to be highly stable in blood and plasma owing to extracellular vesicle carriage and resistant to RNase activity unlike tissue miRNAs [73] and therefore can be considered as valuable potential biomarkers for diagnostic and prognostic purposes. Clinical validation and standardization of methodology [74] can enable the future clinical translation of circulating miRNAs for risk profiling in subjects with periodontitis for preeclampsia and for therapeutic application.

## 5. Conclusion

Nine shared circulatory DEmiRNAs were identified as candidate linkage epigenetic mechanisms between periodontitis. Epigenetic deregulation of target genes MAFB, PSAP, and CDK5RAP2; KEGG pathways renin-angiotensin system and graft-versus-host disease; GO terms “positive regulation of epidermal growth factor-activated receptor activity” and “sequestering of calcium ion”; and target gene-related pathways associated with sirtuins, Wnt/B-catenin, and PPAR signaling were evident as the most important candidate epigenetic mechanisms. These data provide a preliminary basis for experimental research investigating epigenetic mechanisms involved in the association of periodontitis with preeclampsia.

## Figures and Tables

**Table 1:** The 9 shared circulatory DEmiRNA in periodontitis with experimentally validated circulating miRNA in preeclampsia.

miRNA	Preeclampsia	Periodontitis	
	*Experimental validation*	*FDR-adjustedpvalue*	*Log fold change*
Hsa-mir-122	Downregulated	0.03	-1.05
Hsa-mir-1301	Dysregulated	0.03	-1.08
Hsa-mir-130b	Upregulated	0.001	-1.29
Hsa-mir-135b	Dysregulated	0.02	1.63
Hsa-mir-149	Dysregulated	0.004	-1.37
Hsa-mir-152	Downregulated	0.005	-1.01
Hsa-mir-210	Dysregulated	0.035	0.96
Hsa-mir-223	Dysregulated	0.03	0.94
Hsa-mir-27a	Dysregulated	0.01	1.32

**Table 2:** 32 target genes overrepresented in the shared DEmiRNA.

Gene	*p* value adjusted	*Q* value	Observed/expected ratio
MAFB	0.006	0.006	85.400
PSAP	0.006	0.006	29.197
CDK5RAP2	0.017	0.017	189.779
XIST	0.017	0.017	142.334
CALD1	0.017	0.017	113.867
PPP6R2	0.017	0.017	113.867
APC	0.017	0.017	47.444
ZNF618	0.017	0.017	38.818
STARD13	0.017	0.017	35.583
HCCS	0.017	0.017	30.500
CCT6A	0.017	0.017	29.448
NPTX1	0.017	0.017	16.502
BIRC5	0.017	0.017	14.598
PARP1	0.017	0.017	14.598
SP3	0.024	0.024	94.889
BNIP3	0.027	0.027	81.333
NCAM1	0.027	0.027	81.333
TXN	0.027	0.027	81.333
CYB5D1	0.027	0.027	23.722
SCYL3	0.045	0.045	18.565
WASL	0.045	0.045	9.901
ATN1	0.045	0.045	56.933
DNAAF5	0.045	0.045	56.933
SPRY2	0.045	0.045	56.933
RAB14	0.045	0.045	17.429
AGPAT3	0.045	0.045	51.758
PPARG	0.045	0.045	51.758
SIPA1L3	0.045	0.045	51.758
WNT1	0.045	0.045	51.758
RMND5A	0.048	0.048	16.113
DYNC1H1	0.049	0.049	47.444
WASHC2C	0.049	0.049	47.444

**Table 3:** Overrepresented KEGG pathways among shared DEmiRNA associated with periodontitis and preeclampsia.

KEGG pathway	*p* value adjusted	Observed/expected ratio
Renin-angiotensin system	0.03	5.59
Graft-versus-host disease	0.02	5.05
African trypanosomiasis	0.03	4.14
Fat digestion and absorption	0.05	3.56
Inflammatory bowel disease (IBD)	0.02	3.43
Valine, leucine, and isoleucine degradation	0.03	3.34
Homologous recombination	0.03	3.32
Hippo signaling pathway-multiple species	0.03	3.21
Ovarian steroidogenesis	0.03	2.87
Hematopoietic cell lineage	0.03	2.66
PPAR signaling pathway	0.02	2.64
Cholesterol metabolism	0.03	2.59
Basal cell carcinoma	0.03	2.33
Phosphatidylinositol signaling system	0.05	1.99

**Table 4:** Overrepresented GO terms among shared DEmiRNA associated with periodontitis and preeclampsia.

Subcategory	*p* value adjusted	Observed/expected ratio
GO0045741 positive regulation of epidermal growth factor-activated receptor activity	0.03	15.35
GO0051208 sequestering of calcium ion	0.01	13.59
GO0016272 prefoldin complex	0.03	12.42
GO0035371 microtubule plus end	0.01	10.87
GO0030877 beta catenin destruction complex	0.04	9.66
GO0009798 axis specification	0.04	9.32
GO0007064 mitotic sister chromatid cohesion	0.03	9.06
GO0030858 positive regulation of epithelial cell differentiation	0.04	9.00
GO0034383 low-density lipoprotein particle clearance	0.04	9.00
GO0070062 extracellular vesicular exosome	0.04	9.00
GO0071837 hmg box domain binding	0.03	8.81
GO0034236 protein kinase a catalytic subunit binding	0.05	8.42
GO0044295 axonal growth cone	0.05	8.42
GO0060770 negative regulation of epithelial cell proliferation involved in prostate gland development	0.05	8.42
GO0051010 microtubule plus end binding	0.03	8.36
GO0046716 muscle cell homeostasis	0.04	7.58
GO0046627 negative regulation of insulin receptor signaling pathway	0.02	7.53
GO0071277 cellular response to calcium ion	0.04	7.41
GO0000718 nucleotide excision repair DNA damage removal	0.04	7.25
GO0007091 mitotic metaphase anaphase transition	0.04	7.09
GO0046676 negative regulation of insulin secretion	0.04	6.39
GO0042157 lipoprotein metabolic process	0.04	6.27
GO0007052 mitotic spindle organization	0.05	5.72
GO0070830 tight junction assembly	0.05	5.72
GO0005881 cytoplasmic microtubule	0.02	5.64
GO0060041 retina development in camera type eye	0.04	5.51
GO0007409 axonogenesis	0.02	5.31
GO0048009 insulin-like growth factor receptor signaling pathway	0.04	4.89
GO0001102 RNA polymerase ii activating transcription factor binding	0.04	4.77
GO0071230 cellular response to amino acid stimulus	0.04	4.66
GO0000724 double-strand break repair via homologous recombination	0.04	4.55
GO0000776 kinetochore	0.03	4.35
GO0090090 negative regulation of canonical Wnt receptor signaling pathway	0.05	4.30
GO0032869 cellular response to insulin stimulus	0.04	4.15
GO0008083 growth factor activity	0.03	3.75
GO0016568 chromatin modification	0.05	3.46
GO0044255 cellular lipid metabolic process	0.05	3.43
GO0030335 positive regulation of cell migration	0.04	3.09
GO0008286 insulin receptor signaling pathway	0.04	2.87
GO0046777 protein autophosphorylation	0.04	2.87
GO0007155 cell adhesion	0.05	2.82
GO0005794 Golgi apparatus	0.04	2.40
GO0008134 transcription factor binding	0.04	2.39
GO0005887 integral to plasma membrane	0.04	2.37

**Table 5:** Significantly enriched WikiPathways.

Name	Adjusted *p* value	Odds ratio	Combined score
NAD metabolism, sirtuins, and aging WP3630	0.01	147.84	1317.13
Regulation of Wnt/B-catenin signaling by small molecule compounds WP3664	0.01	88.68	710.29
Ciliary landscape WP4352	0.01	13.31	105.01
Wnt/beta-catenin signaling pathway in leukemia WP3658	0.02	55.40	395.97
The influence of laminopathies on Wnt signaling WP4844	0.02	40.27	263.85
Photodynamic therapy-induced HIF-1 survival signaling WP3614	0.02	37.97	244.55
Ectoderm differentiation WP2858	0.02	15.20	100.12
Breast cancer pathway WP4262	0.02	13.58	85.18
Wnt signaling pathway WP363	0.04	26.56	153.16
The overlap between signal transduction pathways that contribute to a range of LMNA laminopathies WP4879	0.04	25.05	141.71

**Table 6:** Significantly enriched BioCarta metabolic and signaling pathways.

Name	Adjusted *p* value	Odds ratio	Combined score
Basic mechanism of action of PPARa, PPARb(d), and PPARg and effects on gene expression Homo sapiens h pparPathway	0.05	161.00	777.86
Overview of telomerase RNA component gene hTerc transcriptional regulation Homo sapiens htercPathway	0.05	107.32	482.58
Visceral fat deposits and the metabolic syndrome Homo sapiens h vobesityPathway	0.05	91.99	401.40
Role of PPAR-gamma coactivators in obesity and thermogenesis Homo sapiens h ppargPathway	0.05	80.48	341.79
How does salmonella hijack a cell Homo sapiens h salmonellaPathway	0.05	64.38	260.59
B cell survival pathway Homo sapiens h bcellsurvivalPathway	0.05	53.65	208.26
Effects of calcineurin in keratinocyte differentiation Homo sapiens h calcineurinPathway	0.05	53.65	208.26
Role of PI3K subunit p85 in regulation of actin organization and cell migration Homo sapiens h cdc42racPathway	0.05	42.91	157.77
Y branching of actin filaments Homo sapiens h actinYPathway	0.05	42.91	157.77
Sprouty regulation of tyrosine kinase signals Homo sapiens h spryPathway	0.05	33.87	117.08
Induction of apoptosis through DR3 and DR4/5 death receptors Homo sapiens h deathPathway	0.05	29.25	97.08
Caspase cascade in apoptosis Homo sapiens h caspasePathway	0.05	29.25	97.08
Segmentation clock Homo sapiens h hesPathway	0.05	27.97	91.68
Multistep regulation of transcription by Pitx2 Homo sapiens h pitx2Pathway	0.05	24.74	78.23
WNT signaling pathway Homo sapiens h wntPathway	0.05	22.18	67.85
Inactivation of Gsk3 by AKT causes accumulation of b-catenin in alveolar macrophages Homo sapiens h gsk3Pathway	0.06	16.92	47.43
HIV-1 Nef: negative effector of Fas and TNF Homo sapiens h HivnefPathway	0.08	12.35	30.98

## Data Availability

The datasets utilized in the present study are publically available.

## Supplementary Materials

Supplementary Materials Supplementary Table S1: circulating DEmiRNAs (adjusted *p* value < 0.05) in periodontitis (GSE31568). Supplementary Table S2: circulating miRNAs experimentally validated in preeclampsia.
