# Supplementary material for: Circulating miRNAs as Epigenetic Mediators of Periodontitis and Preeclampsia Association
Source: Dis Markers. 2022 Jul 11;2022:2771492. doi: 10.1155/2022/2771492 (PMC9293528; doi:10.1155/2022/2771492)
Supplement: Supplementary Materials — Supplementary Table S1: circulating DEmiRNAs (adjusted p value < 0.05) in periodontitis (GSE31568). Supplementary Table S2: circulating miRNAs experimentally validated in preeclampsia. [file 2771492.f1.pdf]

Supplementary Table S1: Circulating DEmiRNAs (adjusted P value <0.05) in Peiodontitis (GSE31568)

ID	FDR adjusted P value	Log fold Change
hsa-miR-608	0.0001	3.8580
hsa-miR-150	0.0005	1.8223
hsa-miR-567	0.0005	3.0893
hsa-miR-130b	0.0005	-1.2851
hsa-miR-301b	0.0010	-1.1932
hsa-miR-758	0.0010	2.7725
hsa-miR-890	0.0014	-1.7445
hsa-miR-564	0.0018	-1.1696
hsa-miR-361-5p	0.0019	1.7082
hsa-miR-661	0.0019	2.6894
hsa-miR-1245	0.0019	-1.4893
hsa-miR-663	0.0019	-1.1924
hsa-miR-10a*	0.0025	2.4449
hsa-miR-1228	0.0025	2.7107
hsa-miR-371-3p	0.0025	2.5073
hsa-miR-1252	0.0025	2.4913
hsa-miR-1294	0.0029	2.4939
hsa-miR-125a-3p	0.0035	2.3114
hsa-miR-1182	0.0035	2.5412
hsa-miR-604	0.0036	-0.9863
hsa-miR-149*	0.0042	-1.3721
hsa-miR-30b*	0.0042	2.5061
hsa-miR-936	0.0044	-1.1207
hsa-miR-630	0.0054	-1.6598
hsa-miR-555	0.0055	-1.4407
hsa-miR-765	0.0055	2.5146
hsa-miR-574-5p	0.0055	1.3771
hsa-miR-152	0.0055	-1.0956
hsa-miR-623	0.0055	-1.0293
hsa-miR-146b-3p	0.0055	-1.2124
hsa-miR-647	0.0060	-0.8588
hsa-miR-191	0.0061	0.5908
hsa-miR-615-3p	0.0061	2.2338
hsa-miR-744	0.0068	1.3246
hsa-miR-340*	0.0070	2.1039
hsa-miR-654-3p	0.0074	2.2696
hsa-miR-514	0.0081	-1.4459
hsa-miR-376c	0.0093	-1.0596
hsa-miR-1295	0.0093	-1.1296
hsa-miR-1293	0.0093	2.2459

hsa-miR-27a	0.0093	1.3196
hsa-miR-500*	0.0093	-0.9336
hsa-miR-138-2*	0.0104	-1.2467
hsa-miR-25*	0.0104	-1.0918
hsa-miR-509-5p	0.0114	-1.6264
hsa-miR-208a	0.0115	-1.1983
hsa-miR-1255b	0.0115	-0.9769
hsa-miR-1231	0.0115	-1.1062
hsa-miR-499-5p	0.0115	-1.5990
hsa-miR-30a*	0.0120	2.0528
hsa-miR-331-5p	0.0127	2.2579
hsa-miR-32*	0.0128	2.1265
hsa-miR-1281	0.0128	2.5557
hsa-miR-1285	0.0129	-1.0657
hsa-miR-646	0.0130	-1.1731
hsa-miR-1228*	0.0130	-1.0143
hsa-miR-425*	0.0130	-1.1418
hsa-miR-379*	0.0130	-1.1437
hsa-miR-448	0.0133	-1.2172
hsa-miR-875-3p	0.0136	-1.2928
hsa-miR-1226*	0.0139	-1.2449
hsa-miR-187	0.0141	1.8879
hsa-miR-590-5p	0.0149	-0.7639
hsa-miR-10b	0.0149	-0.9386
hsa-miR-370	0.0149	1.8858
hsa-miR-593*	0.0149	-0.9486
hsa-miR-451	0.0149	1.2369
hsa-miR-518f	0.0152	-1.2092
hsa-miR-10b*	0.0161	-1.0831
hsa-miR-571	0.0168	-1.0688
hsa-miR-187*	0.0170	1.4510
hsa-miR-153	0.0170	-0.9316
hsa-miR-513b	0.0170	1.9438
hsa-miR-410	0.0171	-1.1225
hsa-miR-133b	0.0171	1.8912
hsa-miR-95	0.0171	-1.6002
hsa-miR-941	0.0171	-0.5551
hsa-miR-875-5p	0.0171	1.9036
hsa-miR-509-3p	0.0171	1.9488
hsa-miR-485-5p	0.0171	-1.1992
hsa-miR-9*	0.0180	1.5571
hsa-miR-592	0.0185	1.9507
hsa-miR-1256	0.0188	-0.9715
hsa-miR-1469	0.0189	-0.8533
hsa-miR-1908	0.0190	-1.1315
hsa-miR-622	0.0196	1.8444

hsa-miR-1267	0.0206	1.9466
hsa-miR-1277	0.0206	-0.9851
hsa-miR-206	0.0206	1.9253
hsa-miR-921	0.0213	-0.9935
hsa-miR-891a	0.0213	-0.7434
hsa-miR-504	0.0213	1.8828
hsa-miR-1205	0.0213	-0.9606
hsa-miR-575	0.0214	-0.7681
hsa-miR-518d-5p	0.0220	-0.9349
hsa-miR-522	0.0224	1.7510
hsa-miR-363*	0.0225	-0.9377
hsa-miR-220b	0.0231	-1.3002
hsa-miR-628-5p	0.0231	-0.8885
hsa-miR-1255a	0.0233	-1.2793
hsa-miR-654-5p	0.0233	-0.9531
hsa-miR-450a	0.0233	1.7157
hsa-miR-1271	0.0233	1.0419
hsa-miR-106a*	0.0233	-1.0166
hsa-miR-548f	0.0238	-1.2384
hsa-miR-506	0.0238	-1.2496
hsa-miR-1234	0.0238	0.8889
hsa-miR-135b	0.0238	1.6293
hsa-miR-367	0.0238	-0.6195
hsa-miR-1306	0.0238	1.9215
hsa-miR-1236	0.0243	1.9316
hsa-miR-1266	0.0267	-0.7287
hsa-miR-1206	0.0286	-0.9558
hsa-miR-1258	0.0293	1.6074
hsa-miR-612	0.0294	-0.8315
hsa-miR-510	0.0294	-1.0697
hsa-miR-1250	0.0294	1.9814
hsa-miR-92b*	0.0294	-0.8344
hsa-miR-92a-2*	0.0295	1.7137
hsa-miR-421	0.0295	1.6562
hsa-miR-767-3p	0.0298	-1.0443
hsa-miR-223	0.0298	0.9417
hsa-miR-124	0.0305	-1.0012
hsa-miR-885-3p	0.0315	-0.8056
hsa-miR-488*	0.0321	-0.7644
hsa-miR-125b-1*	0.0322	1.7154
hsa-miR-339-5p	0.0322	0.9007
hsa-miR-933	0.0322	-1.2407
hsa-miR-432	0.0327	1.6014
hsa-miR-501-3p	0.0327	-0.6598
hsa-miR-559	0.0327	-0.8544
hsa-miR-217	0.0327	-1.0151

hsa-miR-154	0.0343	1.5064
hsa-miR-548e	0.0343	-0.9260
hsa-miR-216a	0.0343	-0.8387
hsa-miR-369-5p	0.0343	-1.3171
hsa-miR-1307	0.0343	1.5491
hsa-miR-653	0.0343	1.6990
hsa-miR-1237	0.0343	1.4459
hsa-miR-1301	0.0345	-1.0808
hsa-miR-20a*	0.0346	-0.8707
hsa-miR-122*	0.0346	-1.0492
hsa-miR-618	0.0349	1.8014
hsa-miR-520a-5p	0.0352	-0.7194
hsa-miR-320a	0.0352	-0.4379
hsa-miR-449a	0.0356	1.5412
hsa-miR-637	0.0356	1.6521
hsa-miR-210	0.0356	0.9647
hsa-miR-34a*	0.0362	-0.8799
hsa-miR-520e	0.0362	1.5779
hsa-miR-423-5p	0.0362	-0.5919
hsa-miR-562	0.0362	-1.2981
hsa-miR-500	0.0362	1.4138
hsa-miR-148b*	0.0362	-1.1013
hsa-miR-641	0.0362	-0.8396
hsa-miR-486-3p	0.0371	0.8049
hsa-miR-148a*	0.0388	1.6554
hsa-miR-548a-5p	0.0390	1.6649
hsa-miR-455-3p	0.0398	-0.7721
hsa-miR-143	0.0401	-0.8444
hsa-miR-483-3p	0.0401	1.7458
hsa-miR-1299	0.0427	-1.2840
hsa-miR-939	0.0429	1.6855
hsa-miR-542-3p	0.0433	-0.9584
hsa-miR-135b*	0.0437	-0.7849
hsa-miR-127-5p	0.0440	-1.1766
hsa-let-7b*	0.0442	1.6663
hsa-miR-342-3p	0.0443	-0.4983
hsa-miR-146b-5p	0.0443	1.2055
hsa-miR-1289	0.0443	-0.7678
hsa-miR-431	0.0443	-0.9457
hsa-miR-487b	0.0443	-0.6373
hsa-miR-302e	0.0446	1.6045
hsa-miR-519a*	0.0448	-1.0809
hsa-miR-302a	0.0448	1.4396
hsa-miR-26b*	0.0448	1.4860
hsa-miR-140-3p	0.0448	-0.2847
hsa-miR-491-3p	0.0448	-0.9810

hsa-let-7d	0.0460	1.3486
hsa-miR-1270	0.0461	-1.3362
hsa-miR-182	0.0462	-0.6083
hsa-miR-101*	0.0470	1.6450
hsa-miR-1468	0.0473	1.3545

Supplementary Table S2: Circulating miRNAs experimentally validated in preeclampsia

Experimentally validated miRNA	Reference PMID
hsa-mir-122	29800045
hsa-mir-126	29800045
hsa-mir-146a	29800045
hsa-mir-152	22095477
hsa-mir-222	29381395
hsa-mir-126	25738738
hsa-mir-1301	25064070
hsa-mir-130a	25738738
hsa-mir-135b	25738738
hsa-mir-136	30261165
hsa-mir-142	25738738
hsa-mir-149	25738738
hsa-mir-155	25981845
hsa-mir-155	28700503
hsa-mir-188	25738738
hsa-mir-18a	25738738
hsa-mir-18b	25738738
hsa-mir-203	25738738
hsa-mir-205	25738738
hsa-mir-210	24035613
hsa-mir-210	25981845
hsa-mir-210	28700503
hsa-mir-210	30324123
hsa-mir-222	25981845
hsa-mir-223	25064070
hsa-mir-224	25064070
hsa-mir-224	25738738
hsa-mir-27a	25738738
hsa-mir-296	25981845
hsa-mir-29a	25738738
hsa-mir-29b	25981845
hsa-mir-301a	25738738
hsa-mir-376	30186390
hsa-mir-494	30261165
hsa-mir-495	30261165
hsa-mir-517c	25738738
hsa-mir-518	25738738
hsa-mir-518e	25738738
hsa-mir-519d	25738738
hsa-mir-93	25738738
hsa-mir-98	25981845
hsa-mir-103a-1	22187671
hsa-mir-103a-2	22187671
hsa-mir-103b-1	22187671

hsa-mir-103b-2	22187671
hsa-mir-106b	30277561
hsa-mir-130b	22187671
hsa-mir-181a-1	22187671
hsa-mir-181a-2	22187671
hsa-mir-210	22095477
hsa-mir-24-1	22187671
hsa-mir-24-2	22187671
hsa-mir-26a-1	22187671
hsa-mir-26a-2	22187671
hsa-mir-326	30277561
hsa-mir-342	22187671
hsa-mir-574	22187671
hsa-mir-885	26853698
hsa-mir-17	26339600
hsa-mir-21	28694210
